# De-escalation for Human Papillomavirus-Positive Oropharyngeal Cancer: A Look at the Prospective Evidence

**DOI:** 10.1007/s11912-025-01652-8

**Published:** 2025-02-26

**Authors:** Allen M. Chen

**Affiliations:** Department of Radiation Oncology, University of California, Irvine, Chao Family Comprehensive Cancer Center, 101 The City Drive, Building 23, Orange, CA 92868 USA

**Keywords:** HPV, De-escalation, Oropharyngeal cancer, Head and neck, Chemoradiation

## Abstract

**Purpose of Review:**

Although it is now firmly established that the presence of human papillomavirus (HPV) expression in oropharyngeal cancer is associated with a favorable prognosis, the implications with respect to treatment remain uncertain. However, the recognition that HPV-positive oropharyngeal cancer is exquisitely sensitive to radiation and chemotherapy has raised questions regarding the appropriateness of historical treatment paradigms, and clinical trials have been conducted to assess whether patients can be treated with less intensive regimens. The fundamental goal of de-escalation is to preserve the high rates of cure and survival from traditional approaches while reducing the incidence of both short- and long-term side effects. However, the data reporting on de-escalation is relatively limited.

**Recent Findings:**

While the evidence to date has been promising, the heterogeneity of the published studies particularly with trial design, de-escalation approach, inclusion criteria, and treatment selection has made drawing definitive conclusions difficult. The use of differing endpoints related to disease control and quality of life have also complicated the comparison of trials across the literature.

**Summary:**

Multiple uncertainties continue to exist with respect to the current state of de-escalation for HPV-positive oropharyngeal cancer, and how to consider the growing evidence in the context of clinical decision-making in the future is the subject of this review.

## Introduction

The worldwide incidence of human papillomavirus (HPV)-associated oropharyngeal squamous cell carcinoma has increased dramatically in recent years reaching epidemic-like proportions in developed countries [1]. For patients with disease confined to the head and neck, radiation therapy, either delivered as primary treatment (to 70 Gy) or postoperatively (to 60–66 Gy), is recommended. While the success of this treatment, typically administered as once-daily radiation over the course of 6 to 7 weeks and often combined with cisplatin chemotherapy, has been well-established from a curative standpoint, the associated toxicity can be pronounced [2]. Side effects including dysphagia, xerostomia, mucositis, and/or fibrosis can limit compliance to treatment, adversely affect quality of life, and can be permanent in many survivors [3].

With the recognition over the last several decades that the presence of HPV in oropharyngeal cancer confers an improved prognosis, studies aiming to de-escalate treatment have generated excitement [4]. Given the strong link between HPV and treatment response, the American Joint Committee on Cancer (AJCC) established a new staging system in 2016 specifically for patients diagnosed with HPV-positive oropharyngeal cancer, to account for its favorable prognosis compared to those with HPV-negative disease [5]. The implications of the revised staging system were dramatic as many patients with HPV-positive tumors that had been previously categorized as stage IV were significantly “down staged” to stage II or even stage I cancers. However, how to refine treatment based on HPV status, despite these staging changes, remains uncertain. While the findings from recently published prospective trials have yielded insight into the feasibility of de-escalation for HPV-positive oropharyngeal cancer, numerous questions persist that limit their current utilization.

## The Evidence

### Rationale for De-escalation

The most important benchmark of any de-escalation regimen is to ensure that the high cure rates observed with standard treatment for HPV-positive oropharyngeal cancer are preserved. Assuming that this bar is satisfied, the potential for de-escalation to improve quality of life can then be addressed. In this respect, the attractiveness of reducing the radiation dose is well-understood as the vast majority of side effects associated with historical treatment have been related to this modality. The rationale herein is rooted in the recognition that a strong relationship exists between the incidental dose delivered to uninvolved organs responsible for function and the likelihood of developing side effects such as dysphagia, mucositis, and xerostomia [6]. However, the impact of chemotherapy on treatment tolerance must also be recognized as cisplatin can not only exacerbate radiation-induced side effects but also lead to nephrotoxicity, neurotoxicity, ototoxicity, as well as bone marrow suppression [7]. Given the contributions of both radiation and chemotherapy to the traditionally high rates of toxicity observed in patients treated for oropharyngeal cancer, attempts to reduce the intensity of either or both make sense.

### De-escalation approaches

De-escalation in the setting of HPV-positive oropharyngeal cancer is a term that has become increasingly elusive to define given the varying approaches that have been outlined to date. While strategies that have been investigated in the definitive and postoperative settings have included the upfront reduction of radiation dose and shrinking of the radiation target volume, a considerable amount of evidence also exists reporting on adaptive de-escalation methods in which the planned radiation dose is lowered depending on the initial response to chemotherapy or chemoradiation [8]. Additionally, attempts to replace cisplatin with other less intensive drugs or to eliminate chemotherapy completely from treatment have also been studied [9]. To complicate matters further, combination approaches in which both the radiation and chemotherapy administration are modified have also been proposed [10].

One of the first published experiences on de-escalation for HPV-positive oropharyngeal cancer arose from the University of North Carolina group [11]. In a phase II study, these investigators reported on 44 patients with minimal smoking history were treated to 60 Gy with concurrent weekly intravenous cisplatin at 30 mg/m^2^. Impressively, the 3-year local control, regional control, cause-specific survival, distant metastasis-free survival, and overall survival rates were 100%, 100%, 100%, 100%, and 95%, respectively. In a subsequent analysis, the de-escalation regimen was associated with favorable patient-reported outcomes, with early recovery of quality of life and continued improvement of xerostomia and dysphagia beyond 1-year post-treatment [12].

Prospective trials utilizing a sequential chemoradiation approach to de-escalation have also been published. Investigators from the University of California performed a phase 2 trial treating 45 patients with locally advanced HPV-positive oropharyngeal squamous cell carcinoma with 2 cycles of induction chemotherapy with carboplatin and paclitaxel given 21 days apart, followed by de-escalated radiation to 54 Gy and 60 Gy to complete and partial responders [13]. The progression-free survival at 2 years was found to be 92%, and a significantly improved toxicity profile compared with historical regimens using standard radiation doses was observed. For instance, the gastrostomy-tube dependence rate at 6-months post-radiation and incidence of grade 3 or higher late dysphagia was both zero. As importantly, post-hoc analysis of quality-of-life endpoints and pre- and post-therapy swallow studies showed that de-escalation dramatically improved function [14]. Furthermore, patients treated by de-escalated radiation had decreased weight loss, depression, and opioid usage compared to historical control subjects [15]. A comparable study conducted by the Eastern Cooperative Oncology Group (ECOG) analyzed 51 patients stratified to 54 Gy after achieving a complete clinical response at the primary site to 3 cycles of induction chemotherapy with cisplatin, paclitaxel, and cetuximab [16]. Among these patients treated by de-escalation, the 2-year progression-free survival and overall survival rates were 80% and 94%, respectively. At 12 months, significantly fewer patients treated with 54 Gy had difficulty swallowing solids (40% versus 89%) or had impaired nutrition (10% versus 44%) compared to the cohort of patients treated to 69.3 Gy because they had less than a complete clinical response to induction chemotherapy.

The Optima trial was another phase 2 de-escalation study in which 62 patients with HPV-positive oropharyngeal squamous cell carcinoma were initially treated by induction chemotherapy with 3 cycles of carboplatin and nab-paclitaxel [17]. Patients were then stratified to de-escalated radiation doses of either 45 Gy or 50 Gy (using twice-daily fractionation with a split-course regimen) depending on their response to induction chemotherapy and their initial risk category based on T-stage and smoking history. Notably, patients with less-than-ideal response were treated to 75 Gy. With this adaptive design, the overall 2-year progression-free survival was 95%. More recently, the results of Optima 2 which aimed to evaluate the efficacy of nivolumab with induction chemotherapy were published suggesting that the incorporation of a checkpoint inhibitor into the treatment backbone may facilitate the selection of subsequent local therapy [18]. The Quarterback trial was another randomized phase 3 study that directly compared reduced dose radiation to standard dose radiation after induction chemotherapy for locally advanced HPV-positive oropharyngeal cancer patients [19]. After 3 cycles of docetaxel, cisplatin and 5-fluorouracil induction chemotherapy, patients with a clinical or radiographic complete/partial response were randomized to receive reduced (56 Gy) or standard (70 Gy) dose radiation with weekly carboplatin. Among the 20 patients randomized, the 3-year progression-free survival rates were not significantly different at 88% and 83% for those receiving standard and reduced dose radiation, respectively.

The largest published study to date is from the NRG Oncology Group HN-002 in which 306 patients were randomized in a phase II setting to either 60 Gy with concurrent weekly cisplatin or 60 Gy delivered in an accelerated fashion without chemotherapy [20]. Although the study ambitiously aimed to evaluate the impact of both radiation dose de-escalation and the omission of chemotherapy and thus could be criticized for not having a standard control arm, the findings were nonetheless instructive. What the trial ostensibly showed that chemoradiation is superior to radiation alone for HPV-positive oropharyngeal cancer, it must be kept in mind that all patients were treated to a reduced dose of 60 Gy, which led to some difficulty in drawing definitive conclusions. Additionally, the improvement in outcomes were only slightly in favor of the chemoradiation arm with the 2-year progression free survival rates being 91% and 88% for patients treated with and without chemotherapy, respectively. Notably, the 2-year overall survival was essentially the same at 97% in both arms.

### Interpretive Synthesis

While the evidence to date suggests that the promise of de-escalation to optimize quality of life while maintaining the historically high rates of cure associated with treatment has largely been validated, it is unclear how to reconcile the differing outcomes obtained through varying approaches. In this sense, the proverbial “800-pound gorilla” relates to selection. Notably, many of the earlier de-escalation studies depended on the old (seventh edition) AJCC staging system in determining eligibility rather than the contemporary (eighth edition) used in practice. Relatedly, imbalances in disease burden across studies may have contributed to the differing results. It is also important to recognize that inclusion criteria based on smoking history varied. This is relevant because published data have suggested that the favorable impact of HPV on prognosis is particularly strong for those patients deemed “never” or “minimal” smokers,” with several groups showing that the conferred benefit associated with HPV is attenuated for those with an increased smoking history [4]. While controversy exists regarding how smoking intensity (as well as the impact of quitting) exactly affects prognosis, most de-escalation studies have used a 10 pack-year tobacco history as the cut-off demarcating “minimal” from “heavy.” Research, however, has now shown that this threshold may be imperfect [21]. For instance, Hawkins et al. showed that any smoking history was associated with inferior overall survival for patients with HPV-positive oropharyngeal cancer, suggesting that even traditionally categorized “minimal” smokers might be at higher risk for recurrence than once thought [22].

The influence of selection is particularly relevant in view of prospective data showing that the substitution of cisplatin with less intensive agents such as cetuximab might compromise outcomes in HPV-positive oropharyngeal cancer patients. While generally not considered classic de-escalation trials, they nonetheless have important implications on the possible omission of chemotherapy for this disease. RTOG 1016 conducted in North America randomized 849 patients with HPV-positive oropharyngeal cancer to 70 Gy with high-dose cisplatin or weekly cetuximab and showed inferior overall survival and progression-free survival for the latter compared with the former [9]. These findings were echoed by the European “De-ESCALaTE” trial which randomized 334 patients with locally advanced p16-positive oropharyngeal cancer to 70 Gy with high-dose cisplatin or weekly cetuximab [23]. The investigators showed a significant difference between cisplatin and cetuximab in 2-year overall survival (98% versus 89%) and 2-year recurrence (6% versus 16%) favoring cisplatin. Lastly, the Trans-Tasman Radiation Oncology Group randomized 189 patients to 70 Gy with weekly cisplatin or weekly cetuximab and showed 3-year failure-free survival rates of 93% and 80%, respectively, among patients treated by cisplatin and cetuximab [24]. While these studies included a generally heterogenous group of patients, notably with respect to tumor volume, clinical stage, and smoking history, they suggest that not all patients might be appropriate for de-escalation approaches based on the alteration or elimination of chemotherapy. However, researchers from Japan recently published findings from a phase II trial which enrolled 39 patients treated by radiation alone to 70 Gy in which the 2-year progression-free survival and overall survival was 94% and 100%, respectively [25]. These data demonstrate that some patients can be treated successfully without chemotherapy. Notably, no patients in their study were feeding tube dependent at 1-month post-treatment, and the incidence of grade 3 or higher toxicity was zero.

Lastly, the results of prospective trials on minimally invasive operative techniques using transoral robotic surgery (TORS) have also been heralded as supporting de-escalation for HPV-positive oropharyngeal cancer [26–28]. Eastern Cooperative Oncology Group (ECOG) 3311 was a phase II study in which 495 patients were stratified to varying treatments based on their pathological findings after TORS [26]. Notably, 110 patients with risk factors which would typically make them candidates for adjuvant radiation alone were randomized to either 50 Gy or 60 Gy and were found to have 2-year progression-free survival rates of 95% and 96%, respectively. Similarly, single-arm phase II data from the Mayo Clinic reporting on the use of 30–36 Gy in 20 fractions using twice-daily treatments in combination with chemotherapy after TORS suggests that dose de-escalation might be an effective strategy [27]. Notably, investigators from the University of Pennsylvania have taken a different approach to de-escalation by decreasing the volume of tissue subjected to radiation [28]. In a phase II trial (AVOID), they showed omitting radiation to the primary site after TORS in the setting of favorable pathological features including negative margins preserved the high rates of cure while optimizing function. Among 60 patients enrolled, only 1 experienced a local relapse, and the 2-year overall survival for the entire population was 100%.

Given the number of ongoing prospective clinical trials on de-escalation for HPV-positive oropharyngeal cancer (Table 1), investigational interest in this subject remains strong. Notably, the study design of current trials varies widely, reflecting the relative lack of agreement regarding the optimal means of de-escalation. It is also important to recognize that nearly all clinical trials, similar to the AJCC staging system, have considered p16-positivity to be equivalent to HPV-positivity. As a result, practical questions of how to define p16-positivity with respect to staining pattern and/or intensity are of relevance. Relatedly, studies have now shown that the discordance between p16 and HPV may be significant in oropharyngeal cancer [29]. This is important because it is now established that patients with p16–negative/HPV-positive or p16-positive/HPV–negative disease had a significantly worse prognosis than patients with p16-positive/HPV-positive oropharyngeal cancer, and a significantly better prognosis than patients with p16–negative/HPV-negative oropharyngeal cancer [30]. Thus, the inadvertent inclusion of patients with p16-positive tumors who are actually HPV-negative have the potential to confound the findings of de-escalation trials and lead to confusion in their interpretation.

**Table 1:** Current prospective trials investigation de-escalation for HPV-positive oropharyngeal cancer

Intent	Trial	Institution	Trial design (sample size)	RT details	Primary endpoint
RT, CRT, or SBRT	NCT04900623	Dana Farber	Phase II (n = 145)	RT 70 Gy/35 vs de-escalated dose (NS) ± chemo based on circulating HPV/DNA	2-year PFS
	NCT03799445	MDACC	Phase II (n = 180)	RT 55–60 Gy/30 (reduced field) with concurrent ipilimumab + nivolumab	2-year PFS
	NCT05600842	UC Irvine	Phase II (n = 111)	RT 60 Gy/30 (6 fractions per week) with chemo in select patients	2-year PFS
	NCT03215719	NYU	Phase II (n = 144)	CRT 70 Gy/35 vs de-escalated dose with concurrent cisplatin	2-year PFS
	NCT04667585	Duke	Phase II (n = 120)	CRT 70 Gy/35 vs de-escalated dose based on mid treatment response in lymph nodes	2-year PFS
	NCT05491512	MSKCC	Phase II (n = 121)	CRT 30 Gy/15 with various chemo regimens incorporating hypoxia	2-year LRC
	NCT03323463	MSKCC	Phase II (n = 316)	CRT 70 Gy/35 vs CRT 30 Gy/15 based on hypoxia	2-year LRC
	NCT03416153	Michigan	Phase II (n = 75)	CRT 70 Gy/35 vs CRT 54 Gy with concurrent carboplatin and taxol	1-year LRC
	NCT03952585	NRG Oncology	Phase II (n = 711)	CRT 70 Gy/35 vs nivolumab + RT 60 Gy/30	6-year PFS
	NCT04178174	Montreal	Phase II (n = 106)	SBRT (14 Gy/3) + 40 Gy/20 with concurrent cisplatin versus CRT 70 Gy/33	2-year LRC
Induction chemo	NCT04867330	Fudan	Phase II (n = 46)	Induction chemo (toripalimab + docetaxel + cisplatin); CRT 70 Gy/35 with cisplatin vs RT 60 Gy/30	2-year PFS
	NCT04572100	U-Chicago	Phase I (n = 50)	Induction chemo (paclitaxel + carboplatin) followed by TORS or RT or CRT	HPV-DNA 20 weeks
	NCT05108870	U-Chicago	Phase I/II (n = 98)	Induction chemo (paclitaxel + carboplatin) with tumor vaccines followed by TORS or CRT	2-year DLT and 2-year RR
	NCT02945631	Mount Sinai	Phase II (n = 50)	Induction chemo; CRT 56 Gy/28 vs CRT 50.4 Gy/28	3-year PFS
	NCT04277858	McGill	Phase II (n = 60)	Induction docetaxel + cisplatin followed by TORS ± PORT	2-year PFS
PORT	NCT03396718	Dresden	Phase I (n = 384)	Multiple PORT regimens including 48.5–55 Gy	2-year LRC
	NCT04920344	Rutgers	Phase II (n = 40)	PORT 50 Gy/25 vs PORT 60 Gy/30	MDADI at 2 months
	NCT05119036	Indiana	Phase II (n = 75)	PORT 54 Gy/27 vs PORT 44 Gy/22	2-year DFS
	NCT03729518	Pennsylvania	Phase II (n = 150)	TORS alone or TORS + reduced volume PORT 50 Gy/25	2-year LRC
	NCT04489212	Mayo	Phase I (n = 45)	TORS + standard PORT vs reduced volume PORT	2-year LC
	NCT03875716	Dana Farber	Phase II (n = 111)	PORT 60 Gy vs PORT 46 Gy vs observation	2-year DFS
	NCT04502407	Cedars Sinai	Phase II (n = 40)	PORT with cisplatin: 50 Gy/25 vs 30 Gy/15	2-year PFS
	NCT02215265	PATHOS	Phase III (n = 1100)	Low risk: No PORT; Int Risk PORT 60 Gy/30 vs PORT 50Gy/25; High Risk: PORT 60Gy/30 vs 60Gy/30 with cisplatin	1-year MDADI/OS

NYU, New York University; MSKCC, Memorial Sloan-Kettering Cancer Center; MDACC, MD Anderson Cancer Center; UC, University of California; HPV, human papillomavirus; DNA, deoxyribonucleic acid; RT, radiation therapy; chemo, chemotherapy; CRT, concurrent chemoradiation; SBRT, stereotactic body radiotherapy; PORT, post-operative radiation therapy; TORS, transoral robotic surgery; PFS, progression-free survival; LRC, local–regional control; MDADI, MD Anderson dysphagia index; DFS, disease-free survival; LC, local control; DLT, dose-limiting toxicity; RR, response rate

Ongoing research focusing on refining de-escalation strategies by incorporating biological-translational techniques have the potential to improve selection. The use of cell-free DNA to quantify disease burden and to longitudinally monitor response as a means of facilitating de-escalation for HPV-positive oropharyngeal cancer is increasingly being studied [31]. Similarly, studies investigating the utility of biological markers such as hypoxia in assessing response and stratifying patients for de-escalation have shown promise [32]. Others have proposed genomic signatures based on immune classifiers or cancer stem cell expression could be useful in stratifying patients for de-escalation [33, 34]. Moreover, the potential of machine learning to facilitate the selection of patients for treatment has been demonstrated [35]. Clearly, advancements in de-escalation will occur as improvements in selection become realized.

## Conclusion

Given its potential to preserve quality of life and function while maintaining high rates of cure, de-escalation has emerged as an attractive option in the management of HPV-positive oropharyngeal cancer. Fittingly, multiple prospective trials have been published in the last decade investigating de-escalation approaches for HPV-positive oropharyngeal cancer. While these studies have generally been encouraging, it is increasingly evident that diverging strategies are emerging which require thoughtful discourse to reconcile how to best make sense of the evidence. Understanding the subtleties that underlie the biological and clinical discrepancies amongst HPV-positive oropharyngeal carcinomas will likely be critical to optimizing treatment strategies in the future. Based on the growing evidence, as well as on the explosion in interest from patients and physicians alike, further research is urgently needed to better refine selection criteria for de-escalation and to stratify patients with newly diagnosed HPV-positive oropharyngeal cancer into the appropriate means of treatment.

## Key References

- Chen AM, Felix C, Wang PC, et al. Reduced-dose radiotherapy for human papillomavirus-associated squamous cell carcinoma of the oropharynx: a single-arm, phase 2 study. Lancet Oncol 2017; 18: 803–811.
  - One of the earliest prospective studies evaluating the feasibility and efficacy of radiation dose reduction for HPV-positive oropharyngeal cancer.
- Marur S, Li S, Cmelak AJ, et al. E1308: Phase II trial of induction chemotherapy followed by reduced-dose chemotherapy followed by reduced-dose radiation and weekly cetuximab in patients with HPV-associated resectable squamous cell carcinoma of the oropharynx- ECOG-ACRIN Cancer Research Group. J Clin Oncol 2017; 35: 490–497.
  - The first published cooperative group study on de-escalation for HPV-positive oropharyngeal cancer.
- Yom SS, Torres-Saavedra P, Caudell JJ, et al. Reduced dose radiation therapy for HPV-associated oropharyngeal carcinoma (NRG 002). J Clin Oncol 2021; 39: 956–965.
  - The largest published series reporting on de-escalation using both radiation dose reduction and chemotherapy omission for HPV-positive oropharyngeal cancer.

## Data Availability

There was no original data generated.

## References

[1] Lechner M, Liu J, Masterson L, et al. HPV-associated oropharyngeal cancer: epidemiology, molecular biology, and clinical management. Nat Rev Clin Oncol. 2022;19:306–27. 35105976 10.1038/s41571-022-00603-7 PMC8805140

[2] Lassen P, Huang SH, Su J, et al. Treatment outcomes and survival following definitive (chemo)radiotherapy in HPV-positive oropharynx cancer: Large-scale comparison of DAHANCA vs PMH cohorts. Int J Cancer. 2022;150:1329–40. 34792199 10.1002/ijc.33876

[3] Van der Laan HP, Van den Bosch L, Schuit E, et al. Impact of radiation-induced toxicities on quality of life of patients treated for head and neck cancer. Radiother Oncol. 2021;160:47–53. 33892023 10.1016/j.radonc.2021.04.011

[4] Ang KK, Harris J, Wheeler R, et al. Human papillomavirus and survival of patients with oropharyngeal cancer. N Engl J Med. 2010;363:24–35. 20530316 10.1056/NEJMoa0912217 PMC2943767

[5] Cramer JD, Hicks KE, Rademaker AW, et al. (2018) Validation of the eighth edition American Joint Committee on Caner staging system for human papillomavirus-associated oropharyngeal cancer. Head Neck 2018;40: 457–466. 10.1002/hed.24974 28990257

[6] Hayakawa T, Kawakami S, Soda I, et al. Dosimetric factors associated with long-term patient-reported outcomes after definitive radiotherapy of patients with head and neck cancer. Radiat Oncol. 2019;14:221. 31818301 10.1186/s13014-019-1429-3 PMC6902539

[7] Furgan M, Snyders TP, Saglain MU, et al. Comparing high-dose cisplatin with cisplatin-based combination chemotherapy in definitive concurrent chemoradiation setting for locally advanced head and neck squamous cell carcinoma. Cancer Med. 2019;8:2730–9. 30968604 10.1002/cam4.2139 PMC6558467

[8] Cmelak AJ, Ferris RL, Chen AM, et al. Treatment de-intensification for HPV-positive oropharynx cancer: what is currently acceptable? J Clin Oncol. 2021;39:2732–3. 34043410 10.1200/JCO.21.00594

[9] Gillison ML, Trotti AM, Harris J, et al. Radiotherapy plus cetuximab or cisplatin in human papillomavirus-positive oropharyngeal cancer (NRG Oncology RTOG 1016): a randomised, multicentre, non-inferiority trial. Lancet. 2019;393:40–50. 30449625 10.1016/S0140-6736(18)32779-X PMC6541928

[10] Rosenberg AJ, Agrawal N, Pearson A, et al. Risk and response adapted de-intensified treatment for HPV-associated oropharyngeal cancer: OPTIMA paradigm expanded experience. Oral Oncol. 2021;122: 105566. 34662771 10.1016/j.oraloncology.2021.105566 PMC9295443

[11] Chera BS, Amdur RJ, Tepper JE, et al. Mature results of a prospective study of deintensified chemoradiotherapy for low-risk human papillomavirus-associated oropharyngeal squamous cell carcinoma. Cancer. 2018;124:2347–54. 29579339 10.1002/cncr.31338

[12] Pearlstein KA, Wang K, Amdur RJ, et al. Quality of life for patients with favorable-risk HPV-associated oropharyngeal cancer after de-intensified chemoradiotherapy. Int J Radiat Oncol Biol Phys. 2019;103:646–53. 30395903 10.1016/j.ijrobp.2018.10.033

[13] Chen AM, Felix C, Wang PC, et al. Reduced-dose radiotherapy for human papillomavirus-associated squamous cell carcinoma of the oropharynx: a single-arm, phase 2 study. Lancet Oncol. 2017;18:803–11. 28434660 10.1016/S1470-2045(17)30246-2 PMC6488353

[14] Hegde JV, Shaverdian N, Daly ME, et al. Patient-reported quality-of-life outcomes after de-escalated chemoradiation for human papillomavirus-positive oropharyngeal carcinoma: findings from a phase 2 trial. Cancer. 2018;124:521–9. 29044458 10.1002/cncr.30954 PMC5916816

[15] Hegde JV, Shaverdian N, Felix C, et al. Functional outcomes after de-escalated chemoradiation therapy for human papillomavirus-positive oropharyngeal cancer: secondary analysis of a phase 2 trial. Int J Radiat Oncol Biol Phys. 2018;100:647–51. 29246721 10.1016/j.ijrobp.2017.10.045

[16] Marur S, Li S, Cmelak AJ, et al. E1308: Phase II trial of induction chemotherapy followed by reduced-dose chemotherapy followed by reduced-dose radiation and weekly cetuximab in patients with HPV-associated resectable squamous cell carcinoma of the oropharynx- ECOG-ACRIN Cancer Research Group. J Clin Oncol. 2017;35:490–7. 28029303 10.1200/JCO.2016.68.3300 PMC5455313

[17] Seiwert TY, Foster CC, Blair EA, et al. OPTIMA: a phase II dose and volume de-escalation trial for human papillomavirus-positive oropharyngeal cancer. Ann Oncol. 2019;30:297–302. 30481287 10.1093/annonc/mdy522

[18] Rosenberg AJ, Agrawal N, Juloori A, et al. Neoadjuvant nivolumab plus chemotherapy followed by response-adaptive therapy for HPV-positive oropharyngeal cancer: OPTIMA II Phase 2 open-label nonrandomized clinical trial. JAMA Oncol. 2024;10:923–31. 38842838 10.1001/jamaoncol.2024.1530 PMC11157444

[19] Misiukiewicz K, Gupta V, Miles BA, et al. Standard of care versus reduced dose chemoradiation after induction chemotherapy in HPV-positive oropharyngeal carcinoma patients: The Quarterback Trial. Oral Oncol. 2019;95:170–7. 31345387 10.1016/j.oraloncology.2019.06.021

[20] Yom SS, Torres-Saavedra P, Caudell JJ, et al. Reduced dose radiation therapy for HPV-associated oropharyngeal carcinoma (NRG 002). J Clin Oncol. 2021;39:956–65. 33507809 10.1200/JCO.20.03128 PMC8078254

[21] Schrank T, Weir W, Lai A, et al. Quantifying smoking exposure, genomic correlates, and related risk of treatment failure in p16+ squamous cell carcinoma of the oropharynx. Laryngoscope Investig Otolaryngol. 2021;6:1376–82. 34938877 10.1002/lio2.695 PMC8665424

[22] Hawkins PG, Mierzwa ML, Bellile E, et al. Impact of American Joint Committee on Cancer eighth edition clinical stage and smoking history on oncologic outcome in human papillomavirus-associated oropharyngeal squamous cell carcinoma. Head Neck 2019; 41: 857–864. 10.1002/hed.25336 PMC6420360 30775826

[23] Mehanna H, Robinson M, Hartley A, et al. Radiotherapy plus cisplatin or cetuximab in low-risk human papillomavirus-positive oropharyngeal cancer (De-ESCALaTE HPV): an open-label randomised controlled phase 3 trial. Lancet. 2019;393:51–60. 30449623 10.1016/S0140-6736(18)32752-1 PMC6319250

[24] Rischin D, King M, Kenny L, et al. Randomized trial of radiation therapy with weekly cisplatin or cetuximab in low-risk HPV-associated oropharyngeal cancer (TROG 12.01) - A Trans-Tasman Radiation Oncology Group study. Int J Radiat Oncol Biol Phys. 2021;111: 876–886. 10.1016/j.ijrobp.2021.04.015 34098030

[25] Takemoto N, Seo Y, Nakahara S, et al. Radiation therapy alone for human papillomavirus-related squamous cell carcinoma of the oropharynx: A single-arm, phase 2 study. Int J Radiat Oncol Biol Phys. 2021;110:403–11. 33373656 10.1016/j.ijrobp.2020.12.025

[26] Ferris RL, Flamand Y, Weinstein GS, et al. Phase II randomized trial of transoral surgery and low-dose intensity modulated radiation therapy in resectable p16+ locally advanced oropharynx cancer: An ECOG-ACRIN Cancer Research Group Trial (E3311). J Clin Oncol. 2022;40:138–49. 34699271 10.1200/JCO.21.01752 PMC8718241

[27] Ma DJ, Price KA, Moore EJ, et al. Phase II evaluation of aggressive dose de-escalation for adjuvant chemoradiotherapy in human papillomavirus-associated oropharynx squamous cell carcinoma. J Clin Oncol. 2019;37:1909–18. 31163012 10.1200/JCO.19.00463 PMC7098832

[28] Swisher-McClure S, Lukens JN, Aggarwal C, et al. A phase 2 trial of alternative volumes of oropharyngeal irradiation for de-intensification (AVOID): Omission of the resected primary tumor bed after transoral robotic surgery for human papilloma virus-related squamous cell carcinoma of the oropharynx. Int J Radiat Oncol Biol Phys. 2020;106:725–32. 31785337 10.1016/j.ijrobp.2019.11.021

[29] Shinn JR, Davis SJ, Lan-Kuhs K, et al. Oropharyngeal squamous cell carcinoma with discordnatn p16 and HPV mRNA results—incidence and characterization in a large, contemporary United States cohort. Am J Surg Pathol. 2021;45:951–61. 33739785 10.1097/PAS.0000000000001685 PMC8192336

[30] Mehanna H, Taberna M, von Buchwald C, et al. Prognostic implications of p16 and HPV discordance in oropharyngeal cancer (HNCIG-EPIC-OPC): a multicentre, multinational, individual patient data analysis. Lancet Oncol 24: 239–251. 10.1016/S1470-2045(23)00013-X 36796393

[31] Chera BS, Kumar S, Beaty BT, et al. Rapid clearance profile of plasma circulating tumor HPV type 16 DNA during chemoradiotherapy correlates with disease control in HPV-associated opharyngeal cancer. Clin Cancer Res. 2019;25:4682–90. 31088830 10.1158/1078-0432.CCR-19-0211 PMC6679766

[32] Lee N, Schoder H, Beattie B, et al. Strategy of using intratreatment hypoxia imaging to selectively and safely guide radiation dose de-escalation concurrent with chemotherapy for locoregionally advanced human papillomarivus-related oropharyngeal carcinoma. Int J Radiat Oncol Biol Phys. 2016;96:9–17. 27511842 10.1016/j.ijrobp.2016.04.027 PMC5035649

[33] Zeng PY, Cecchini MJ, Barrett JW, et al. Immune-based classification of HPV-associated oropharyngeal cancer with implications for biomarker-drive treatment de-intensification. eBiomedicine 2022; 86: 104373. 10.1016/j.ebiom.2022.104373 PMC9706534 36442320

[34] Kristensen MH, Sorensen MK, Tramm T, et al. Tumor volume and cancer stem cell expression as prognostic markers for high-dose- loco-regional failure in head and neck squamous cell carcinoma—a DAHANCA 19 study. Radiother Oncol. 2024;193:110149. 38341096 10.1016/j.radonc.2024.110149

[35] Alabi RO, Almangush A, Elmusrati M, et al. An interpretable machine learning prognostic system for risk stratification in oropharyngeal cancer Int J. Med Inform. 2022;168:104896. 10.1016/j.ijmedinf.2022.104896 36279655

